# Correction: Effects of hemodynamic load on cardiac remodeling in fish and mammals: the value of comparative models

**DOI:** 10.1242/jeb.249924

**Published:** 2024-12-06

**Authors:** Jared B. Shaftoe, Todd E. Gillis

There was an error in *J. Exp. Biol.* (2024) **227**, jeb247836 (doi:10.1242/jeb.247836).

In Fig. 2, structural remodeling in the red-eared slider turtle heart following cold acclimation was indicated to increase compact layer thickness. This was misinterpreted from figure 5 of Keen et al. (2016) (doi:10.1152/ajpregu.00510.2015) which shows an increase in collagen content in the compact layer.

**Fig. 2.**
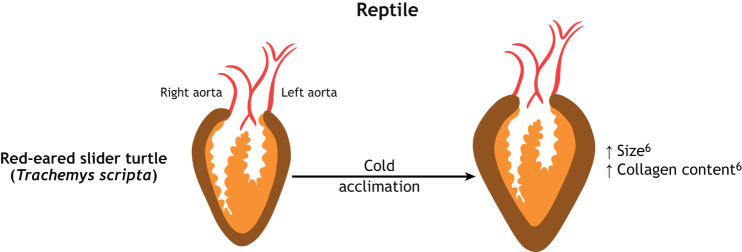
**(corrected panel). The effect of thermal acclimation on the morphology and composition of hearts in two teleost fish and a reptile.** Red-eared slider turtle (*Trachemys scripta*). Compact thickness is shown in brown, bulbous arteriosus, atrium and aorta are shown in red and spongy myocardium is shown in orange; collagen content is not shown. ^6^Keen et al. (2016b).

Keen et al. (2016) did not directly measure the size of the myocardial layers. To correct this error, the line indicating the increase in compact layer thickness has been removed from Fig. 2. The corrected and original versions of this panel are shown below.

Likewise, in Table 2, remodeling of the turtle heart after cold acclimation was stated to increase compact layer thickness. This cell in the table has been removed.

**Fig. 2.**
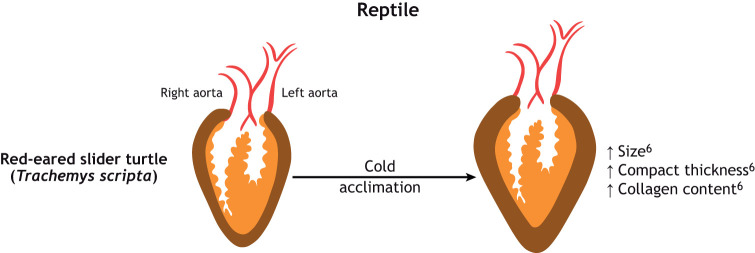
**(original panel). The effect of thermal acclimation on the morphology and composition of hearts in two teleost fish and a reptile.** Red-eared slider turtle (*Trachemys scripta*). Compact thickness is shown in brown, bulbous arteriosus, atrium and aorta are shown in red and spongy myocardium is shown in orange; collagen content is not shown. ^6^Keen et al. (2016b).

Both the online full text and PDF versions of the paper have been corrected. The authors sincerely apologize to readers for these errors.

